# Adenocarcinoma Arising From a Cervical Esophageal Inlet Patch: The Malignant Potential of a Small Lesion

**DOI:** 10.7759/cureus.9284

**Published:** 2020-07-19

**Authors:** Karolina N Dziadkowiec, Sergio A Sánchez-Luna, Peter Stawinski, Jose Proenza, Eduardo P Delaflor-Weiss

**Affiliations:** 1 Internal Medicine, University of Miami, John F. Kennedy Regional Campus, Atlantis, USA; 2 Center for Advanced Therapeutic Endoscopy/Division of Gastroenterology, Hepatology and Nutrition, Allegheny Health Network/Allegheny Center for Digestive Health, Pittsburgh, USA; 3 Gastroenterology, West Palm Beach Veterans Affairs Medical Center, West Palm Beach, USA; 4 Pathology, West Palm Beach Veterans Affairs Medical Center, West Palm Beach, USA

**Keywords:** cervical inlet patch, endoscopy, esophageal adenocarcinoma

## Abstract

Inlet patches (IP) are heterotopic lesions consisting of gastric mucosa. Commonly located in the cervical esophagus, it is believed that they are remnants of fetal columnar epithelium arising from incomplete replacement during embryogenesis. A rare complication of IP is the development of proximal esophageal adenocarcinoma. We report a case of a 59-year-old male with intractable cough and dysphagia that was found to have a malignant transformation of an IP.

## Introduction

Esophageal adenocarcinomas typically develop through the metaplasia-dysplasia-carcinoma sequence. Adenocarcinoma within the proximal esophagus, in the location of an inlet patch (IP) and unrelated to Barrett’s metaplasia, is extremely rare. The cervical IP exists in an estimated 3.8%-10% of the general population [1]. IPs are congenital gastrointestinal anomalies and have been reported to occur anywhere along the gastrointestinal system [2]. The presence of this ectopic tissue has been associated with gastroesophageal reflux disease (GERD) symptoms, esophageal and extraesophageal syndromes, bleeding, ulcer formation and even malignant transformation [3-6]. Lesions are often located distal to the upper esophageal sphincter and can be overlooked during routine endoscopy. Similarly, many endoscopists may not be aware of this condition, causing it to be underreported in many instances. 

## Case presentation

A 59-year-old Caucasian man with a history of heart failure, obesity, and tobacco abuse presented to the emergency department two years prior to current presentation, with a five-month history of non-productive cough, bilateral neck pain, globus sensation, and progressive dysphagia. 

**Figure 1:**
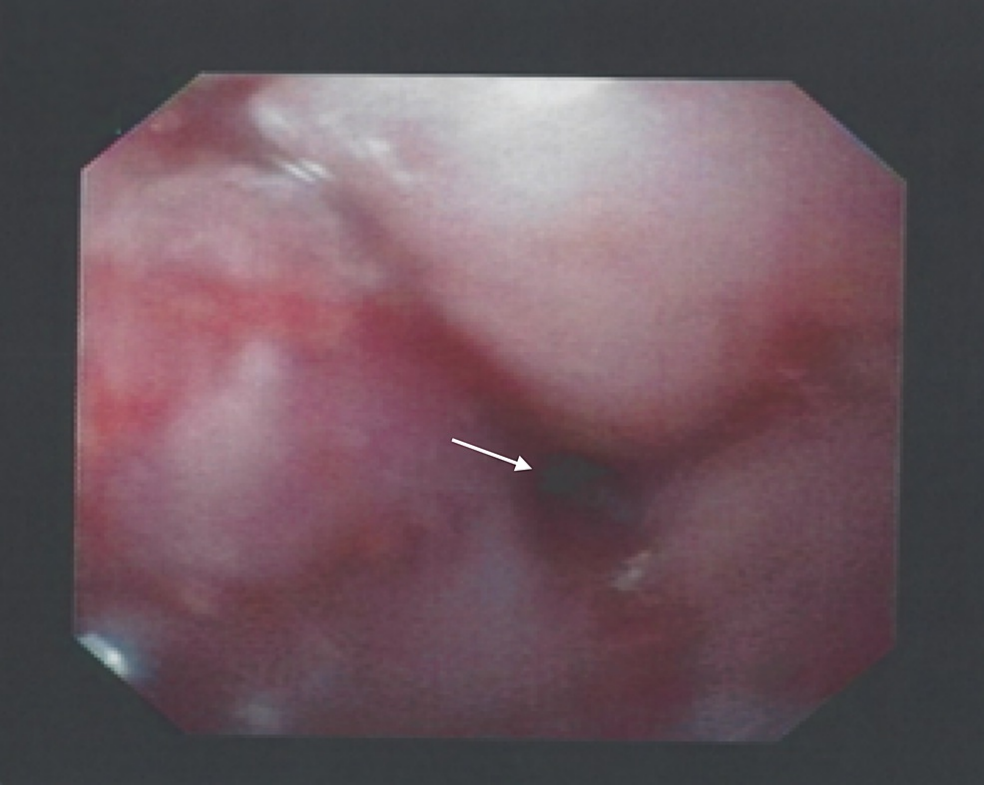
Esophagogastroduodenoscopy: Initial endoscopic findings showing a smooth non-obstructive stricture at the level of the cervical esophagus.

Barium swallow at that time showed an irregularity at the distal cervical esophagus and further visualization on esophagogastroduodenoscopy (EGD) revealed a smooth non-obstructive stricture extending 17 to 20 cm from the incisors (Figure 1). Biopsies from this stricture were negative for metaplasia, dysplasia, or malignancy, and thus the patient was deemed to have an esophageal IP. Complete blood count, metabolic profile, lactate dehydrogenase, and hepatic function were normal. 

**Figure 2:**
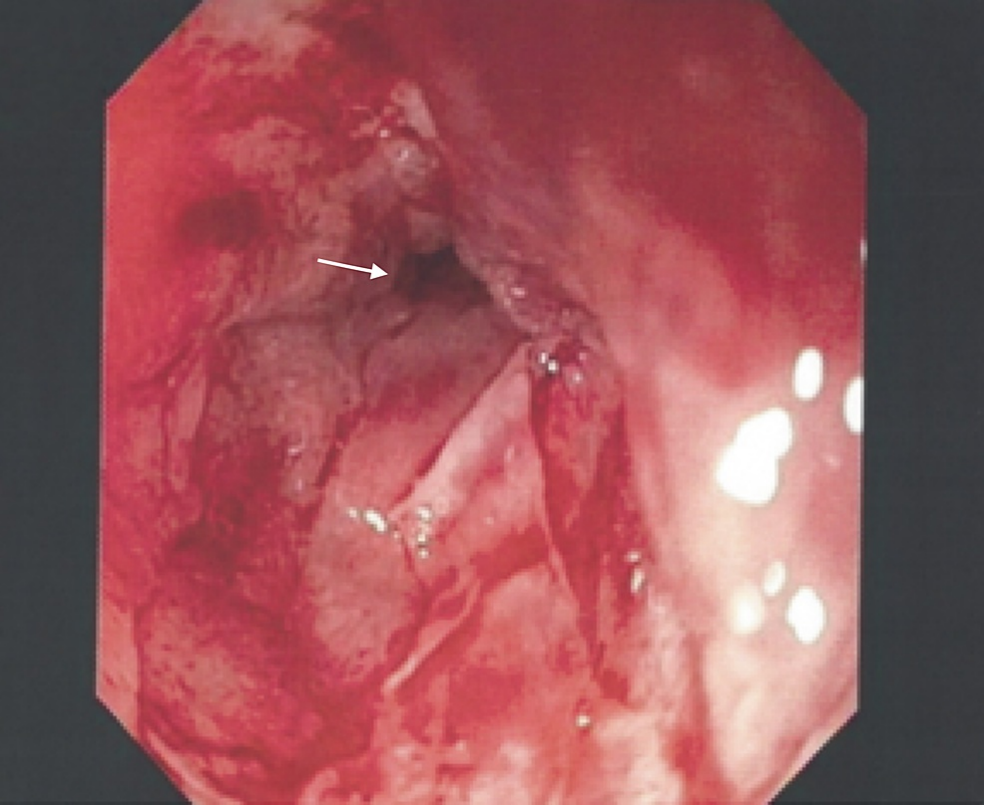
Repeat endoscopic findings in the cervical esophagus revealing a malignant-appearing stricture.

**Figure 3:**
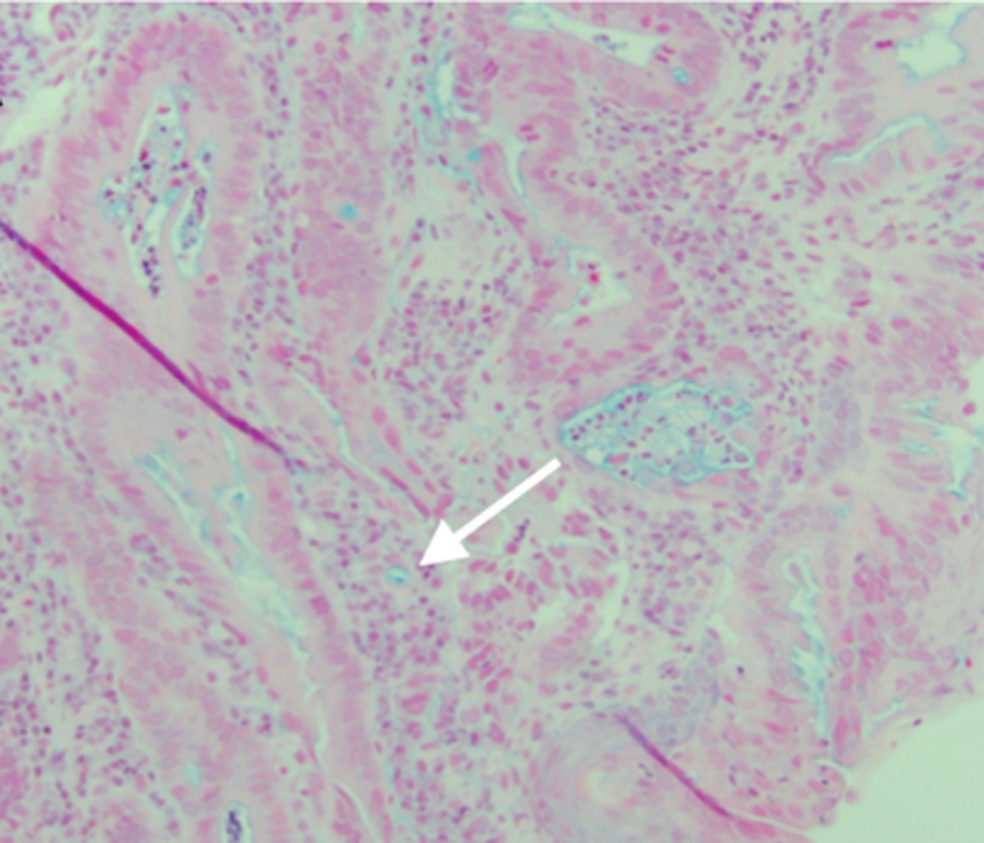
Histopathology of biopsy specimen. Adenocarcinoma of cervical esophagus arising from a patch of gastric-type mucosa. The arrow points to adenocarcinoma with intestinal-type goblet cells.

**Figure 4:**
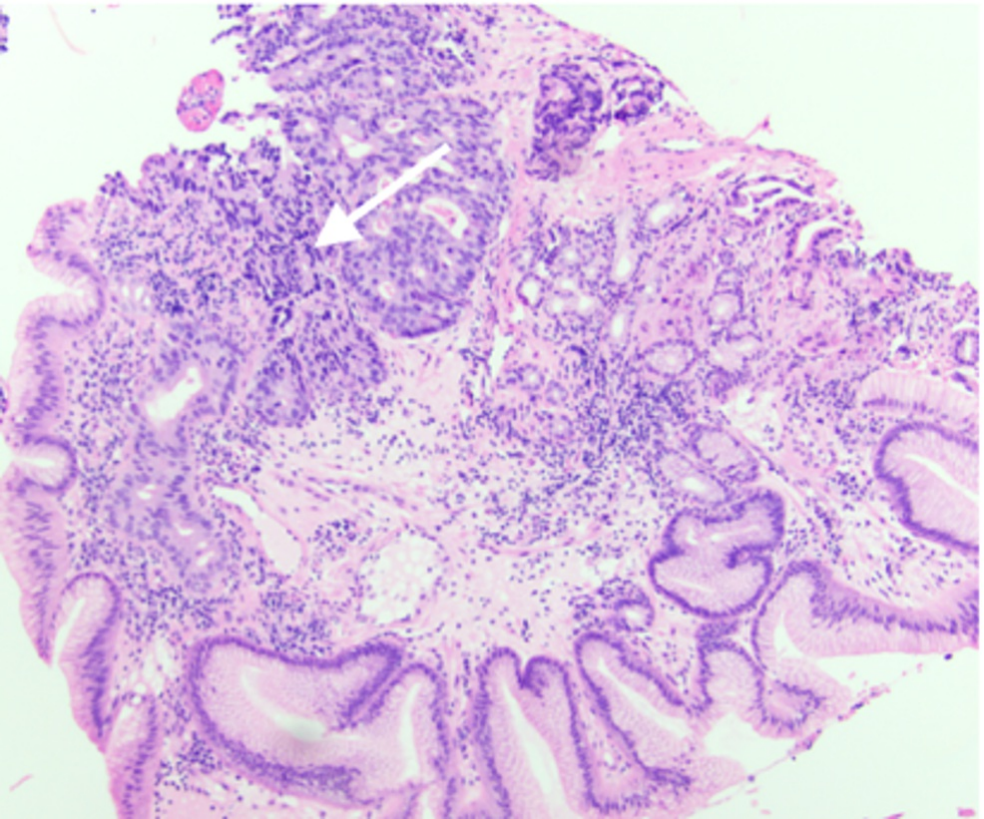
Histopathology of biopsy specimen. Adenocarcinoma of gastric-type mucosa (white arrow).

The patient was subsequently lost to follow-up and returned two years later, complaining of progressive and worsening dysphagia. Repeat EGD was performed (Figure 2) and biopsies revealed adenocarcinoma (CK7+, CK20-, and variable staining for CDX2) of the cervical esophagus, invading at the lamina propria, arising from ectopic gastric mucosa of the previously observed IP during the initial EGD (Figures 3, 4). The patient was deemed a poor surgical candidate and was treated with palliative carboplatin and paclitaxel with adjuvant radiation therapy. At two months of follow-up, the patient continues to be doing clinically well. 

## Discussion

Heterotopic gastric mucosa located of the upper esophagus (HGMUE) is also known as a cervical IP and was first described by Schumidt in 1805 [7,8]. The pathogenesis is widely considered to be congenital, although some studies suggest that metaplastic transformation may also be possible [9]. Their prevalence varies from less than 1% to 14%. Most IPs are asymptomatic and are discovered incidentally during endoscopy, but rarely they may initially present as reflux, esophagitis, stricture, and ulcer formation (due to acid production in the ectopic mucosa or Helicobacter pylori infection of the ectopic mucosa) and produce symptoms consistent with chest or throat pain, dysphagia, or globus esophagus [3-6,10]. The incidence of adenocarcinoma arising from a cervical IP is reported to be 1.5% or less [11]. Proximal esophageal adenocarcinoma arising from ectopic gastric tissue is extremely rare, with less than 60 case reports during current literature review. The pathogenesis of these tumors is poorly understood, and most cases are believed to arise sporadically or through the metaplasia-dysplasia pathway secondary to recurring chemical trauma. Our case is unique since it describes a pathway for the development of adenocarcinoma within the IP which could have included factors, such as locally secreted acid inducing the metaplasia-dysplasia pathway or the intrinsic development of adenocarcinoma within the focus. Endoscopy images showed no continuity of non-malignant mucosa with the stomach. In addition, the absence of goblet cells on pathology indicates a high likelihood that this malignancy originated from the cervical IP. 

Currently, there are no guidelines to guide the management of incidentally discovered IPs. Symptomatic patients are often treated with proton pump inhibitor therapy or ablative therapy with radiofrequency ablation or argon plasma coagulation [12]. Some studies recommend routine endoscopic surveillance, although prospective data have failed to demonstrate long-term benefits, and thus this is currently not recommended. With mounting evidence, it is clear that IPs carry significant clinical importance due to their malignant potential. This case demonstrates the need for increased awareness in identifying and managing these lesions discovered during routine endoscopy, especially when patients develop new or worsening symptoms on follow-up [13].

## Conclusions

Cervical esophageal IPs are commonly observed but rarely biopsied for evidence of dysplasia. Also, IPs can be easily overlooked, and thus careful examination of the cervical esophagus is always warranted. Although it appears that malignant progression is rare, it is important that endoscopists be aware of this potentially dangerous possibility. Recognition of IPs at endoscopy may identify patients at greater risk of developing adenocarcinomas of the proximal esophagus; however, this relationship and the necessity for screening require more study in larger populations.
