# Correction: Adenocarcinoma Arising From a Cervical Esophageal Inlet Patch: The Malignant Potential of a Small Lesion

**DOI:** 10.7759/cureus.c74

**Published:** 2022-09-13

**Authors:** Karolina N Dziadkowiec, Sergio A Sánchez-Luna, Peter Stawinski, Jose Proenza, Eduardo P Delaflor-Weiss

**Affiliations:** 1 Internal Medicine, University of Miami, John F. Kennedy Regional Campus, Atlantis, USA; 2 Center for Advanced Therapeutic Endoscopy/Division of Gastroenterology, Hepatology and Nutrition, Allegheny Health Network/Allegheny Center for Digestive Health, Pittsburgh, USA; 3 Gastroenterology, West Palm Beach Veterans Affairs Medical Center, West Palm Beach, USA; 4 Pathology, West Palm Beach Veterans Affairs Medical Center, West Palm Beach, USA

This article has been corrected to include Eduardo P. Delaflor-Weiss as last author. Dr. Delaflor-Weiss was unintentionally omitted by the submitting author due to an "oversight." The authors deeply regret this error.

